# RESTful M2M Gateway for Remote Wireless Monitoring for District Central Heating Networks

**DOI:** 10.3390/s141222447

**Published:** 2014-11-27

**Authors:** Bo Cheng, Zesan Wei

**Affiliations:** State Key Laboratory of Networking and Switching Technology, Beijing University of Posts & Telecommunications, Beijing 100876, China; E-Mail: weizs.bupt@gmail.com

**Keywords:** RESTful M2M gateway, Wireless remote monitoring, Programmable logic controller, district heating

## Abstract

In recent years, the increased interest in energy conservation and environmental protection, combined with the development of modern communication and computer technology, has resulted in the replacement of distributed heating by central heating in urban areas. This paper proposes a Representational State Transfer (REST) Machine-to-Machine (M2M) gateway for wireless remote monitoring for a district central heating network. In particular, we focus on the resource-oriented RESTful M2M gateway architecture, and present an uniform devices abstraction approach based on Open Service Gateway Initiative (OSGi) technology, and implement the resource mapping mechanism between resource address mapping mechanism between RESTful resources and the physical sensor devices, and present the buffer queue combined with polling method to implement the data scheduling and Quality of Service (QoS) guarantee, and also give the RESTful M2M gateway open service Application Programming Interface (API) set. The performance has been measured and analyzed. Finally, the conclusions and future work are presented.

## 1. Introduction

In recent years, the increased interest in energy conservation and environmental protection, combined with the development of modern communication and computer technology, has resulted in the replacement of distributed heating with central heating in urban areas. Central heating is gradually becoming the primary source of heating in the winter. Compared with the distributed heating used previously, central heating has the advantages of saving energy, reducing pollution, and improving economic efficiency. Central heating is also an important symbol of urban modernization. District heating networks, together with water and gas networks, belong to the class of pipe networks. Pipe networks themselves are part of general infrastructure networks that range from transportation, telecommunication to electricity [1]. Therefore, the establishment of a safe, stable, and expandable monitoring system for the heating network is important for the effective overall management of a central heating network [2,3]. The study of the application of communication technology to heating pipe networks helps improve the level of modern management of urban heating networks [4]. To automate district central heating and reduce human involvement, it is necessary to develop a remote monitoring and control system that monitors the boilers and heating exchange station and can reduce the mistakes caused by workers. The Programmable Logic Controller (PLC) is the core of the boilers and the heating exchange station control system [5]. PLCs are mainly used for the internal storage of instructions to implement such functions as logic, sequencing, timing, counting, and arithmetic to control the digital or analog input/output modules for various types of machine processes.

There are many applications of different strategies for the monitoring and control of district heating systems. The OLE for Process Control (OPC) server [6] is applied in the urban heat supply monitoring and control system which can connect between the devices and HMI software via GPRS channel. Wireless infrastructure in a district heating substation has been proposed [7], and the wireless network uses TCP/IP all the way to the sensor level, which can enable a standardized Service Oriented Architecture (SOA) and the possibility to upgrade the software of each node remotely over the Internet. A distributed district heating substation monitoring system is proposed in [8] for supervising the consumption of customers and the parameters in every substation belonging to a district heating system as well as the weather parameters. The acquired data are transfer on-line across the Internet to a database on a web server. A multi-agent system for distributed control of district heating systems is proposed in [9], which can control the district heating system using demand-side-management strategies dynamically. The upgrading of the district heating system through the Wireless Sensor Networks (WSN) is proposed in [10], where a pilot project conducted by Riga Heat (the main heating supplier in Riga, Latvia) has allowed gaining real life experience in a district in-house heating substation. To enable a cost effective monitoring process, a deployment of a wireless monitoring system for indoor comfort assessment in a smart heat load-shifting context is proposed in [11], which allows a rapid deployment of the monitoring system. Control method based on a fuzzy cognitive map and its application in district heating networks is proposed in [12], which proposes a method utilizing δ learning rules to realize the intelligent control of the district heating supply process.

The communication network is the contact hub of the whole heating monitoring control system, through which heating transfer station, heat source, monitoring nodes form a unified whole. General Packet Radio Service (GPRS) [13,14] allows users to send and receive data at end-to-end packet transfer mode, without requiring the use of circuit-switched mode network resources, thus providing an efficient, low-cost wireless packet data services. Especially suitable for intermittent, sudden and frequent, small amounts of data transmission, but also for the occasional large amount of data transmission. The theoretical bandwidth of GPRS is up to 171.2 Kbit/s, and the practical bandwidth is about 40–100 Kbit/s, GPRS provides TCP/IP connection, can be used for Internet access, data transmission and other applications.

Machine-to-machine (M2M) [15,16] communications is a new concept, wherein effective application devices and problem-solving solutions for the communication of humans, mobile devices, or machines with machines are developed to achieve remote monitoring. For example, for data transfer from M2M, data that are distributed over many places can be collected through a data collection terminal and then delivered through a wireless transfer. The data control center terminal is configured with a dedicated module to unify the data collection and process the data. Then, using wireless transfer, control commands are sent to each data terminal. The M2M technique does not require manual intervention and can automatically upload data, improving the efficiency of information processing. Using Web technology, the Web of Things (WoT) [17,18] implements the abstraction, the integration, and the availability of all types of resources, such as data and applications, located at various sensor nodes in the web environment. Web-based access to resources and services is provided through the WoT. Representational State Transfer (REST) [19,20] is a series of guidelines to meet the Web standards presented in a distributed architecture software style. The RESTful M2M gateway provides the data driver and implements the Uniform Resource Identifier (URI) specification design for all the resources on the server. The external data service is provided with the REST interface, remote access and user control of the M2M gateway server, including the mapping relationship between the user access query and the M2M gateway server. The data exchange between the M2M gateway server system and the sensor or wireless sensor networks includes the acquisition and control of the data in the sensor.

In this paper, we present a RESTful M2M gateway for wireless remote monitoring of a district central heating network. The RESTful M2M gateway can monitor and control the physical sensor devices deployed in the heating exchange stations and boiler rooms. The innovation and contributions of this study are as follows:

(1) Propose a resource-oriented architecture for the RESTful M2M gateway. From the perspective of the structure, the RESTful M2M gateway is divided into three layers, *i.e.*, the device resource abstraction layer, resource management and control layer, and open service interface layer.
(2) Propose a uniform devices abstraction framework, which adopts the OSGi technology to create a uniform protocols management framework with the Dependence Inversion Principle to access the different physical devices.
(3) Propose and implement the resource address mapping mechanism between RESTful resources and the physical sensor devices, also present the buffer queue combined with polling to implement the data traffic congestion and scheduling algorithm.
(4) Design the RESTful M2M gateway open service API access interface, and define the API set for remote wireless monitoring and control for district central heating network.

The rest of the paper is organized as follows: Section 2, describes the RESTful M2M gateway architecture. In Section 3, we describe the experiments and performance measurements. The conclusions and future work are given in Section 4.

## 2. Architecture

**Figure 1.**
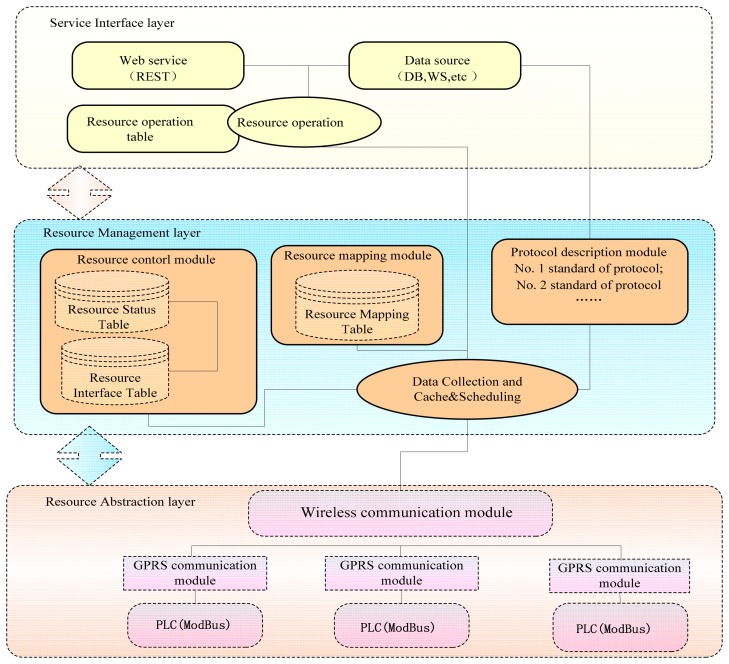
RESTful M2M architecture.

The gateway bridges that connect physical sensing networks with traditional communication networks, M2M gateways can realize the conversion between different types of sensor networks and different types of communication networks. M2M gateways can also realize such functions as data forwarding, protocol conversion, and control management. The structure of the RESTful M2M gateway system can be divided into three layers, *i.e.*, device resource abstraction layer, resource management and control layer, and service interface layer. The RESTful M2M gateway adopts a resource-oriented architecture, which uses the URI design of the gateway resources. The structure is shown in Figure 1.

### Resource abstraction layer

The main functions of resource abstraction are to shield the differences between physical devices (including the differences between devices in terms of implementation details and the differences between transmission protocols), and to provide with a unified framework for obtaining the device data and operating various devices, which adopts the OSGi technology to create a uniform protocols management framework with the Dependence Inversion Principle to access the different physical devices.

### Resource management layer

Resource control management is the core functions for realizing the M2M gateway, which includes the registration, resource management, physical devices configuration management, log management, event management, data processing and storage and data acquisition. Also the data traffic congestion and scheduling algorithm is implemented.

### Open service interface layer

Open service interface is to encapsulate various types of resource in an M2M gateway or capabilities provided by the M2M gateway to form Web services for applications. The M2M gateway can provide the data and capabilities to the terminal device, which is connected to the gateway, and can support M2M applications for directly reading device data and sending control commands through a REST interface on the Internet.

When the gateway receives the data request sent by the client, it parses the data, matches the data with those in the resource operation table, and completes the positioning operation. Then, the resource-mapping module implements the mapping between the application level and the physical level for the resource, *i.e.*, mapping to the physical address in the physical module. By searching and matching the resource status description table and the interface description table in the resource management module, the data request is converted to a standard protocol data packet, and the data packet is issued to a GPRS communication module through the established data path. When the communication module receives the data request packet, and sends a reply packet to the gateway. The gateway parses this reply packet, converts it to realistic and effective data, and then updates the status of the system resource. Additionally, it encapsulates the information with a specified data format and returns it to the client. Thus, the client receives the monitoring and control of the sensor data.

## 2.1. M2M Gateway Resource Abstraction Layer

### 2.1.1. M2M Gateway Data Acquisition Protocol

The sensors store the data in real time in different storage areas of the PLC device through physical wiring, and the PLC device is connected to the communication module with physical wires. The communication module has the network communication function and implements the data communication with the Internet. The Modbus protocol [21] is a serial communications protocol specification used in industrial control, and the Modbus protocol used in the hardware module produced by different companies can vary. Enterprises such as Samsung, Siemens, and Delta all produce different PLC devices. Modbus protocol has two different modes of operation, *i.e.*, the remote terminal unit (RTU) mode and the American Standard Code for Information Interchange (ASCII) mode. These two working modes are described in the following:

*RTU mode*, when the controller is set to communicate in the RTU mode on the Modbus network, every 8-bit byte in the information contains two 4-bit hexadecimal characters. The primary advantage of this mode is that under the same baud rate, more data can be transferred in the RTU mode than in the ASCII mode.

*ASCII mode*, when the controller is set to communicate in ASCII mode on the Modbus network, every 8-bit byte in the information is transmitted as an ASCII code (two hexadecimal characters). The primary advantage of this approach is that the time interval for character transmission can be as long as 1 s without generating an error. If we use the PLC in the ASCII mode, when the data packet is being sent, except for the start of the text character (STX) and the end, the first four bits and the last four bits of every byte are converted to ASCII code. For example, if a byte represents 5A, it is converted to two characters, “5” and “A”. The conversion format is shown in Table 1.

The longitudinal redundancy check (LRC) in the data packet is a verification model that represents the inspection calculation for the data in the data packet. The verification procedure is the following. The adaptive dynamic routing (ADR) is summed to the LRC. In the summation, the byte is the smallest unit; when there is carryover in the process of summing, the carryover is ignored; and, eventually, the complement of the sum with respect to 2 is the value of the LRC. The details of the calculation of the LRC verification code is as follows:
**STX****Address****Function****Data1****Data2****Data3****Data4****LRC****End**3A0103040100010D0A

The procedure of computation is as follows: Address + Function + Data1 + Data2 + Data3 + Data4 = 01 + 03 + 04 + 01 + 00 + 01 = 0A; convert the value of 0A to the complement (the complement is the reverse of every bit in the data to be converted, *i.e.*, 0 changes to 1, and 1 changes to 0, and then, the reversed data are added to 1 to derive the complement); and the 2-complement of 0A is F6. For both the transmitting data packet and the receiving reply packet, the data verification is required to ensure that the received data are usable and effective.

#### 2.1.2. M2M Gateway Wireless Communication Protocol

When the M2M gateway communicates with the underlying device, the GPRS protocol is used, which adopts the request-reply mode for data exchange. The gateway transmits the data request packet, and the GPRS communication terminal returns the data reply packet. This communication protocol has a strict data format. Therefore, the data path must be established with the communication module to read and write on the PLC. The PLC not only stores the switch data, such as the open and closed states of valves, but also stores analog data, such as the temperature, the pressure, the power, and the flow velocity. Table 2 shows the analog data (for both the RTU mode and the ASCII mode) in the acquisition of the PLC in the sent Modbus request packet.

When the PLC issues a Modbus data request packet for analog data, it parses the data packet and then encapsulates the acquired data as the reply packet (for both the RTU mode and the ASCII mode) to return to the server. The format of the reply packet is shown in Table 3.

The GPRS communication protocol is a commonly used wireless communication protocol. This communication protocol does not have a unified standard. Therefore, the specific communication protocol used by the GPRS module produced by various companies may be different. However, every GPRS protocol uses a socket connection for data transfer. The Hongdian H7000 protocol is a GPRS communication protocol. In the H7000 protocol, there are strict guidelines for the format of the data and the report. The report includes the head, the packet type, the packet length, the data, the inspection data, and the tail. The packet length includes the length of the entire protocol packet. The protocol packet uses hexadecimal transmission; the high byte is in the front, and the low byte is in the back. The data packet format of the H7000 protocol is as follows:
**Head (0** × **7B)****Type (8 Bits)****Length (16 Bits)****Data****Tail (0** × **7B)**HeadTypeLengthTail

In the H7000 protocol, there are many types of data packets, such as the registration packet of the terminal request, the cancellation packet of the terminal request, and the request for the IP address of a particular desktop unit (DTU). The detailed types of data packets are shown in Table 4.

The M2M gateway system uses the communication module to establish the inter-sensor data transfer. The GPRS communication module will initially send the terminal registration packet to the M2M gateway system. When M2M gateway receives the terminal registration packet, it responds to the terminal registration packet, and the reply with the successful registration is sent to the terminal. When the GPRS communication terminal receives the reply packet, the connection between the communication terminal and the server is established. That is, the registration of the terminal on the server is successful, the data path between the terminal and the server is established, and it can then be used for the exchange of data. The data format for the reply packet returned from the server is as follows:
**Head****Type****Length****DTU ID****Tail**1 byte1 byte2 bytes11 byte1 byte0 × 7B0 × 810 × 100 × 7B

After the data path between the terminal and the M2M gateway is established, the data can be transferred using this data path. The sensor data are stored in the PLC, and the PLC and the GPRS communication module are connected. Therefore, when the PLC interacts with the server end, it encapsulates the server data in the loading region in the GPRS communication protocol. When the server parses the load, it acquires the sensor data. The format of the users' data packet is as follows:
**Head****Type****Length****DTU ID****Client Data****Tail**1 byte1 byte2 bytes11 byte⩽1024 bytes1 byte0 × 7B0 × 890 × 100 × 7B

When the terminal receives the data request package sent from the M2M gateway, it replies to the data request package. The PLC encapsulates the format of the Modbus data protocol used by the sensor required by the server and transmits it to the GPRS module through hard-wiring. The GPRS terminal module encapsulates the Modbus protocol data packet in its user data domain and transmits it to the M2M gateway system through the established data path.

Here, the OSGi technology [22] is adopted to create a uniform protocol management framework with the Dependence Inversion Principle (DIP) to access the different physical devices. Here, the Dependence Inversion Principle means that the high level modules should not depend on low level modules. Both should depend on abstractions, and the abstractions should not depend on details; instead, the details should depend on abstractions. Hence, the physical device protocol components are decoupled, and the interaction depends on the abstract interface. When a new data packet is sent from the sensor device to the protocol stack framework, the protocol management framework will publish this new data packet to the protocol stack bus such that the protocol stacks deployed in the protocol container will be allowed to analyze this new data packet in a top-down order. If the H7000 protocol can analyze this data packet, then it will be loaded to the protocol stack as the lowest layer protocol. After the data packet is analyzed by the H7000 protocol, the extracted data payload will be published to the upper layer protocol. This data payload is in fact the complete data packet of the M-Bus and ModBus protocol. The protocol of this layer will then attempt to analyze this data payload. If ModBus can accurately analyze this data packet, then ModBus will be loaded as the second layer protocol (*i.e.*, the top layer) of the protocol stack. Thus, the dynamic adaptation of the protocol stack is completed. In addition, this protocol stack is bound to the sensor device that sent this data packet; the applications of this protocol stack will be responsible for analyzing future data packets sent from this sensor device.

### 2.2. M2M Gateway Resource Control and Management

#### 2.2.1. Resource Definition

After abstraction, the data in the entire system is labeled as different resources, whereas the server can display different resources according to varying data requests from the client end. Moreover, the resources may exist in a variety of formats, e.g., html, xml, and json. Because the system adopts the standard hypertext transfer protocol (HTTP) method, we do not need to worry about changing the name of the interface. In the RESTful M2M gateway system, the data source must be accessed on the M2M gateway from the client end, and the real-time data inspection and the data control for the sensor occur in the sub-station. In this system, there are many sub-stations connected to the M2M gateway, and every sub-station has many sensors. Additionally, for different sub-stations, there are the same sensors as well as different sensors. Consequently, a hierarchical sensor design is implemented in these production bases. The key to the RESTful M2M gateway architecture is the abstraction of the users' demands as a resource. In general, as the abstraction becomes more accurate, the structure of the entire system becomes clearer. The style of REST architecture is to create, read, update, and delete (CRUD) the resource on the server. Therefore, the resource on the gateway is designed according to the REST architecture. When querying the value of a certain sensor, the PLC number connected by the sensor and the sub-station number of the PLC must both be known. They can be found by abstracting the corresponding resource through the monitoring and control of resources, *i.e.*, by query and modification. In this situation, the definition of resources includes the sub-station information, the PLC information, and the sensor information, as shown in Table 5.

In a system based on REST, the resource is identified with a URL, and every URL represents a resource. The whole system consists of resources, and therefore, the key for the design of a systematic structure is whether the URLs are designed reasonably. URL design does not have a unified norm; however, network applications are usually hierarchical. Therefore, the URL design must also reflect the hierarchy. A hierarchical structure is adopted in the resource quest module. For example, /stations is the collection of production bases, /stations/1 is the sub-station with :id equal to 1, /stations/1/plcs is all the PLC information for the No.1 sub-station, and /stations/1/plcs/1 is the information of the No.1 PLC accessing the No.1 sub-station. The specific resource label is shown in Table 6.

The REST architecture specifies the method to define the application program interface. To facilitate the data transfer between the program on the client end and the server resource, the HTTP application protocol is used to contact the resource, and the GET, PUT, POST, and DELETE methods are used to perform the relevant operations on the resource. A section of the interface for the query operation and the update operation for the resource are shown in Table 7.

According to the simple description of the resource interface in Table 7, the data exchange with the sensor occurs in the bottom layer, *i.e.*, the read and write operations on the sensor, as shown in Table 8.

When the client end sends a message to the server resource, the M2M gateway parses the resource information requested by the URL, and then provides the field information. The procedure is shown in Figure 2.

As shown in Figure 2, after the data access layer receives the data request from the client end, it parses the URL and then parses the field, which may include the sub-station number, the number of the PLC used, and the sensor number. This information is compared with the resource operation table. If it is determined that the operation is legal, all the field information will be encapsulated into a data object, which is then transferred to the resource adaptation layer. If the operation is illegal (e.g., a writing operation to a read-only sensor), an error message will be returned. The resource operation table is the simple regulation and definition of the resources on the server. It specifies the form of resource access for the client end. A simple example of the description of a resource operation table is shown in Table 9.

Table 9 is a simple description of the resource operation table, where stationid is the number of the production base, plcid is the number of the PLC used, sensorid is the number of the sensor, type is the type (where 1 is readable only, and 2 is readable and writable), high is the upper limit, and low is the lower limit. With respect to read-write sensors, when the client end sends a message to the gateway data, the data access layer parses the request of the user and inspects the resource operation description table. If it recognizes that the operation is legal, it will encapsulate it as a data object in XML format, as follows:

<?XML version= “1.0” encoding= “ UTF-8”?><DataService> <StationId>1</StationId> <PlcId>1</PlcId > <SensorId>100</SensorId > <Value>58.5</Value ></DataService>

After the M2M gateway processes these requests, the data are exchanged with the sensor through relevant techniques, and the data required by the client are obtained. These data are encapsulated into an XML format file and then sent back to the client.

#### 2.2.2. Resource Mapping Mechanism

The PLC stores the data collected by the sensor in real time within its storage area. Because different sensors measure different physical variables with different definitions, in the PLC, the measurement of the different sensors is stored in different registers. Therefore, the operation of reading and writing sensor data is implemented on the register specific to each sensor. In the field of industrial control, hardware companies divide the storage area in PLCs into seven domains (*i.e.*, S, X, Y, T, M, C, and D). Storage domains S and M store the switch data (such as the open and closed states of valves and parameter correction on and off states), and the data stored in storage domain D are the analog data (such as temperature, pressure, and wind velocity). Therefore, the form address of the resource must be converted to the physical address.

In Table 10, Device is the storage area region (such as region S or region M). Range is the range of address numbers on the application level, e.g., the range of address numbers for region S is 0–1023. Type is the value represented by the storage area (bit indicates the switch data and word indicates the analog data). The communication address Hex is the specific register address of this storage area in the PLC. For example, the register address of S512 in the storage area of this PLC is 0 × 0200. Therefore, the address information constituted by any Device and Range must be converted to the form in the communication address Hex column. This then facilitates the operation of reading and writing on a specific register in the communication with the sensor. Because different hardware devices have different storage area divisions, the design of a mapping rule table for a specific hardware device can be used to map the abstract address of that device. Using the Delta PLC as an example, the structure of the mapping rule table is shown in Table 11.

For any abstract address (storage area and number), the aforementioned table can be used to find the region in the PLC corresponding to the location of the resource. Then, through the mapping mechanism, the resource is mapped from abstract to concrete. The procedure is shown in Figure 3.

As shown in Figure 3, the procedures to map the resources address are as follows:

(1) Extract the data containing the PLC model and the description used by the resource. Divide the abstract description of the resource into two parts, *i.e.*, the storage area and the number.
(2) According to the PLC model of the resource, load the corresponding mapping rule table.
(3) If the mapping rule table fails to load, stop and return an error message.
(4) After successful loading of the mapping rule table, use the extracted storage area and number to map the specific storage area.
(5) If the mapping fails, stop and return an error message.
(6) If the mapping is successful, perform the address conversion through the appropriate algorithms.
(7) Complete the resource mapping.

The resource can be mapped from the description on the application layer to the description on the physical layer, and to extract the information specific to the sensors for that resource by searching the resource status description table, including the descriptive information of the sensor, the data protocol used by the sensor, and the communication protocol connected to the sensor. The detailed structure of the resource status description table is shown in Table 12.

The structure shown in Table 12 illustrates that one communication module can connect multiple PLCs and that one PLC can connect multiple sensors. The data required by the system are the physical data collected by the sensor, which are stored in the storage area opposite to the PLC and connected through hard-wiring. The sensor has many characteristics, including the sensor number, the definition of the sensor data (e.g., temperature or pressure), the sensor data, and the data units. In addition, there is also the number of the PLC that connects to the sensor and the data protocol used by the PLC. The resource state description table describes the details of the resource information and resource approach in the access system.

The interface description table contains the basic information for the terminal communication module connected to the gateway, including the number of the terminal communication module, the GPRS protocol employed, and the interface number used by the terminal communication module to connect the system. The interface description table depicts the M2M gateway and terminal communication module information. As the bridge, these communication modules provide the channel for data exchange between the bottom sensor and the server. The interface description table structure is shown in Table 13.

The terminal communication module number is unique. In addition, the GPRS protocol used in the terminal communication module is also different because of varying physical modules. The STATUS indicates whether the terminal communication module is usable at present, whereas OPEN indicates whether this module has been connected to the gateway server through the GPRS protocol.

There are many procedures necessary for the resource management to process a data request. The procedures encompass searching in the resource status description table and in the interface information of the query description table. The procedures for data processing in resource management layer are outlined in Figure 4.

(1) Parse the XML data object. Extract the resource position information.
(2) Map the application layer to the physical layer. Identify the specific address of the physical storage.
(3) Search the resource status description table and extract the resource information and the usable condition of the resource. In the resource description table, if the STATUS associated with a resource is 0, the sensor is not usable; if the STATUS is 1, that sensor is usable. Then extract COM_ID from the matching term information. COM_ID is the number of the terminal communication module connecting the sensor to the server; this number is unique.
(4) Search the interface description table using COM_ID to determine if the terminal communication module is usable. If there is no matching term, then there is no terminal module with this number. If there is a matching term, but the STATUS of the matching term is 0, then the terminal communication module is not usable, and an error message is returned.
(5) Extract the resource protocol information. If the terminal communication module is usable, the DEVICE_PROTOCOL information from the matching resource is extracted.
(6) Find the matching protocol information in the protocol description module. If there is a protocol corresponding to DEVICE_PROTOCOL, return the protocol information. If there is no matching term, return an error message.
(7) Encapsulate the data packet according to the specific protocol format.

#### 2.2.3. Data Traffic Congestion and Scheduling

With the continuously increasing number and types of sensors in M2M gateway, the data volumes of sensors in M2M gateways are increasing rapidly, whereas the processing speed and capacity of the middleware of M2M gateways are limited to a certain degree. In addition, due to different sensor types, the data access cycles, data volumes, and requirements for reading and writing are also different. Therefore, it is necessary to define a suitable scheduling algorithm for accessing different sensors, which can increase the efficiency of the communication between an M2M gateway and sensors and save the communication and processing resources of the M2M gateway.

The buffer queue combined with polling to implement the data scheduling, which ensures the real-time performance of the data and service quality. First, for the data cache problem, a data buffer queue is configured for each device to store the data that cannot be processed for the time being. Second, for the data scheduling problem, priority configuration is performed on the buffer queues based on the user-defined parameters in the service quality configuration file, in addition, data scheduling is performed based on the priority levels. The priority configuration of data buffer queues is not simply to assign high, medium, and low priority levels, instead, a hierarchical configuration method is used, and each data buffer queue is expressed by priority level and priority weight attributes. If the priority levels are the same, then the priority weights are compared. The queue with a higher priority weight has the higher priority and will be processed first. The priority weight is obtained from the mapping function of the maximum allowed processing time. For the same priority level, the mapping functions are the same. For different priority levels, the mapping functions are also different.

### 2.3. M2M Gateway Open Service Interface

Remote wireless monitoring for district central heating network is not only can obtain the data from the wireless sensor network using a REST-style API, but also capable of wireless sensor network device control and management via REST-style API, making the physical device loosely coupled. Here, we apply the RESTful design guidelines to define some additional API set to monitor and control the physical devices and instruments in heating exchange station and boiler rooms. Figure 5 summarizes the detailed specification of which methods are allowed for each resource.

Here, we present some monitoring and control automation scenarios of the proposed RESTful APIs. The majority of service requests for controlling and monitoring the devices can be handled in a single invocation:

*Control list of available devices*, an operator can control the list of available physical devices sending a GET request to retrieve the list of possible configurations and use this information in order to access each context (URI: /Resource/<id>).

*Access the current setting of a device*, the parameters of a device represents the current setting of a device. The list can be retrieved using a GET request to /Resource/<id>/Device/<id>/Parameters where /Device/<id> identify a device with configuration /Resource/<id>/.

*Find devices list*, assuming that the URI /Recource/<id>/device/<id> represents the device supervisor, the list of controlled sensors can be retrieved using a GET request to /Resource/<id>/Device/<id>/ that return the list of URIs identifying the devices controlled by the supervisor.

*Find commands of given device*, the URI /Resource/<id>/Device/<id>/ addresses the device that we want to use. We can retrieve the list of available commands sending a GET request at the URI /Resource/<id>/Device/<id>/Command/.

*Execute control command*, we can execute a measurement command sending a POST request at the URI / Resource /<id>/Device/<id>/Command/measure the results of the measure can be fetched sending a GET request to the URI/ Resource /<id>/Device/<id>/Attributes/<id>.

## 3. Experiments and Performance Measurements

### 3.1. Experimental Setup

**Figure 6.**
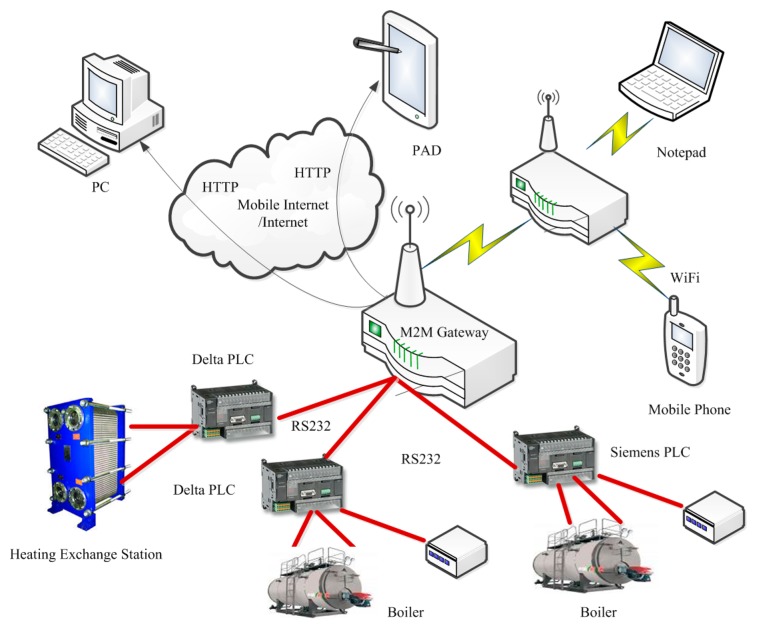
Topology diagram of the test environment.

The experimental setup used to evaluate our M2M gateway is introduced as follows. The RESTful M2M gateway software has been installed on an industrial computer NISE-3600 (Intel^®^ Core™ (Intel, CA, USA) 2.7 G with 2 × DDR3 8 G DDR RAM, Intel^®^ GbE LAN port). The installed software includes the H2 memory database, which is used to store the data tables, and also the open-source Apache Tomcat v.6.0.25 has been installed, which is used to deploy various web services required in the data processing. Around the RESTful M2M gateway, and one Siemens and two Delta PLC devices from different boiler rooms and heating exchange stations are connected via the COM1, COM2, and COM3 serial ports. And the data collection time interval is 5 s. The test environment for the RESTful M2M gateway is shown in Figure 6.

### 3.2. Performance Measurement

#### 3.2.1. Experiment 1: Packet Loss Test

**Figure 7.**
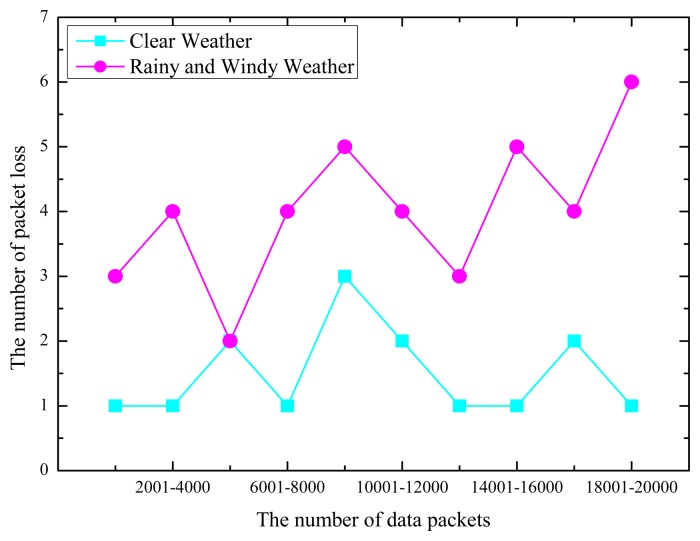
Statistics of data packet loss.

Because the data packet issued by the GPRS module contains the sequence number, we can determine if there is packet loss through the statistics of the sequence number for the received data packets. To perform the test, we deployed a total of 10 GPRS modules to transmit the data with a transmission period of 30 s. When the server collects 2000 data packets, it reports the packet loss statistics. A total of 20 experiments were performed, 10 in clear weather and 10 in windy and rainy weather. The statistical results are shown in Figure 7.

In Figure 7, the horizontal axis marks each of the 10 experiments: every experiment receives 2000 data packets. The vertical axis is the packet loss. The data show that in clear weather, when 20,000 data packets are received, a total of 15 packet loss events occur. The average packet loss rate over 10 experiments is only 0.75%.

In bad weather, when 20,000 data packets are received, a total of 40 packet loss events occur. In this case, the average package loss rate over 10 experiments is 2%. The packet loss rate is relatively low, therefore satisfying the requirement. In Figure 7, the data show that in good weather, the communication is very good; however, when the weather is adverse, communication degrades. We should consider methods to ensure communication quality and reduce the packet loss rate in adverse weather.

#### 3.2.2. Experiment 2: Time Delay Test

**Figure 8.**
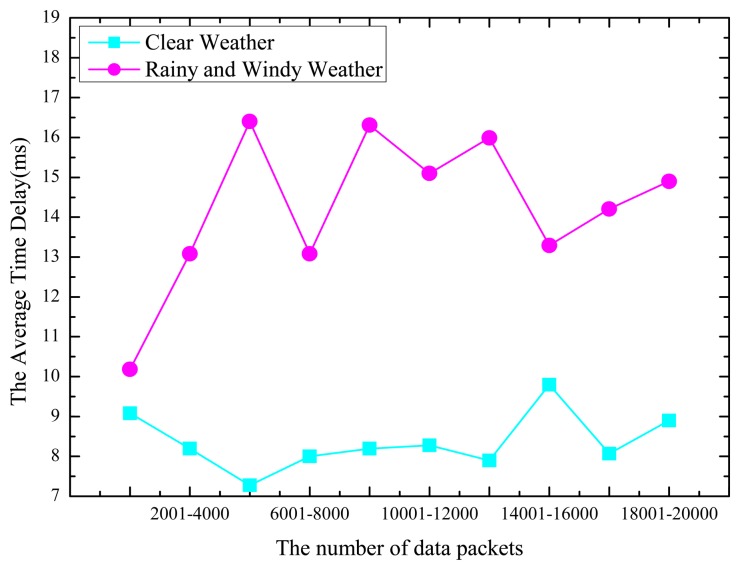
Time delay statistics.

The time delay is the time interval for the server to acquire the data required by the user and return the data to the user through data exchange with the sensor after the user sends a message to the server resource. The bulk of the time is used by the communication delay between the server and the sensor. In this test, we deployed 10 GPRS modules to transmit the data sent by the bottom layer sensor for a period of 30 s. In the course of this experiment, we sequentially record the time delay of the transmission for every time in the file. When the server receives 2000 data packets, it calculates the communication time delay. We conducted a total of 20 experiments. We conducted 10 experiments in clear weather and 10 experiments in windy and rainy weather. The final statistical results are shown in Figure 8.

In Figure 8, the value of the horizontal axis marks each of the 10 experiments, and every experiment receives 2000 data packets. The value of the vertical axis is the average time delay in ms. The data show that, in clear weather, the average time delays for these ten experiments are 9.324691, 8.304323, 7.43123, 8.021342, 8.211280, 8.33452, 7.78127, 9.58279, 8.32645, and 8.78372 ms. In adverse weather, the average time delays for these 10 experiments are 10.5623, 13.3761, 16.4872, 13.2341, 16.3498, 15.3286, 15.9362, 13.637, 14.485, and 14.873 ms. The data in Figure 8 show that when the weather is clear, the average time delay is less than 10 ms, whereas when the weather is bad, the average time delay is over 10 ms, and it is more than 13 ms in most cases. Therefore, we should consider how to reduce communication delays caused by adverse weather. According to the test results above, in conditions of good weather and adverse weather, the packet loss rate and system delays are both in the controllable and receivable range, allowing the system to operate well in any conditions. However, because this system adopts the GPRS communication protocol to acquire and control sensor data, to further ensure normal communication operations, effort should be made to improve the quality of communication, reduce the packet loss rate, and reduce the communication time delays.

#### 3.2.3. Experiment 3: Data Cache and Scheduling

**Figure 9.**
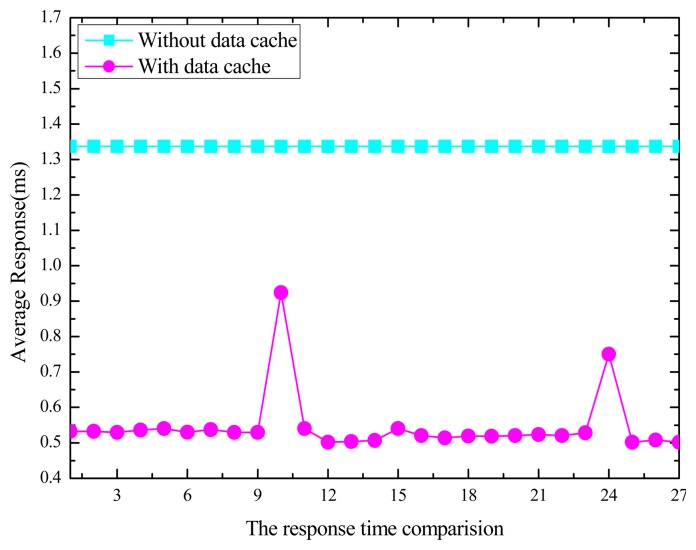
Response time for user requests.

One or more clients' requests are applied to collect the data from the M2M gateway devices with data cache function and without data cache function separately, and the response time for user requests was tested. Figure 9 shows the experimental results.

As Figure 9 shows, the performance test of the M2M gateway before and after modification the data cache and polling function, and the response time of the M2M gateway for user requests significantly decreased from 1.3 s (original mean value) to 0.5 s.

#### 3.2.4. Experiment 4: Response Time for Concurrent Threads

**Figure 10.**
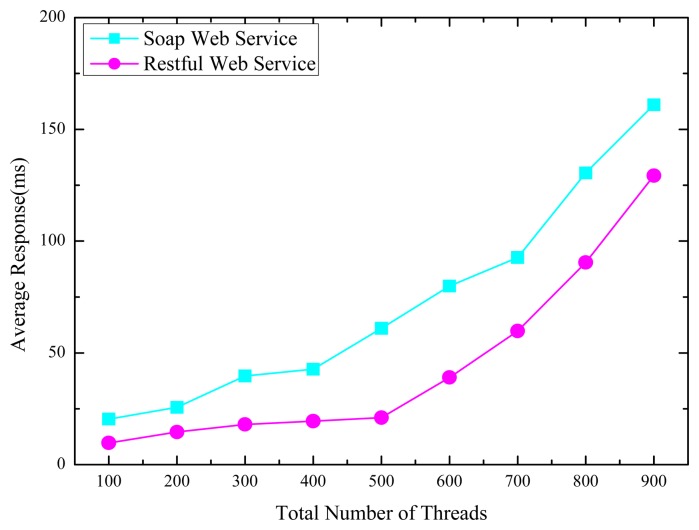
Average response times for concurrent threads.

One or more clients' threads are applied to invoke the same remote control service with RESTful API and SOAP Web service interface respectively, and the value of the client threads is automatically generated and configured. The goal of this experiment is to understand the scalability, the overhead and the flexibility our proposed RESTful APIs compared with the existing SOAP based Web service implementation. The response time for both SOAP and RESTful based Web services are shown as Figure 10.

As Figure 10 shows, the SOAP based Web service consumes much more response time. Considering the average response times, the RESTful Web service outperforms SOAP based Web service as expected.

## 4. Conclusions

In this paper, we propose a RESTful M2M gateway for remote wireless monitoring of district central heating networks, and M2M gateway is used to collect the monitoring data from the heating exchange station and boiler rooms and send it to a master station via a wireless GPRS channel. Firstly, we propose a resource-oriented architecture for the RESTful M2M gateway, and focus on the uniform devices abstraction approach, REST resources mapping mechanism and the data traffic congest and QoS guarantee, and design of the open service API access interface. Also, the RESTful M2M gateway for remote wireless monitoring for district central heating network has been deployed in the heating exchange stations and boiler rooms in Beijing KingFore Energy-Saving Technology Co., Ltd (Beijing, China). In the future, because of there are lots of types of protocols for electrical equipment, the M2M gateway will be extended to suit more protocols, and the performance should be tested in larger scale deployment environment.

## Figures and Tables

**Figure 2.**
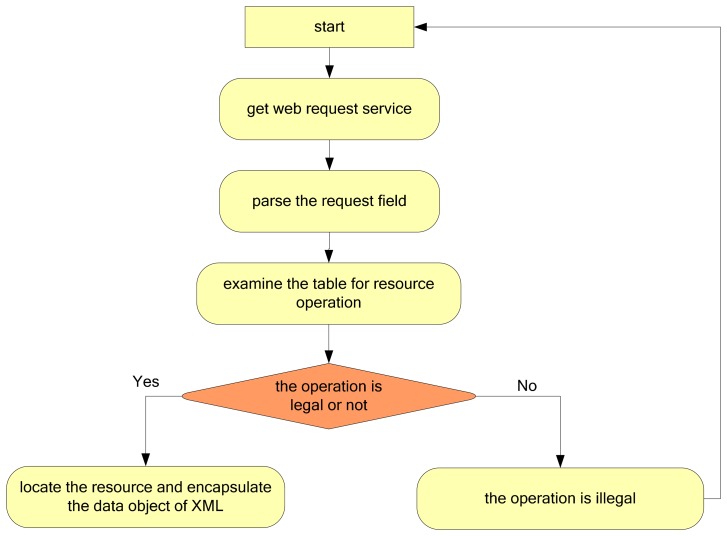
Flowchart to handle the data request.

**Figure 3.**
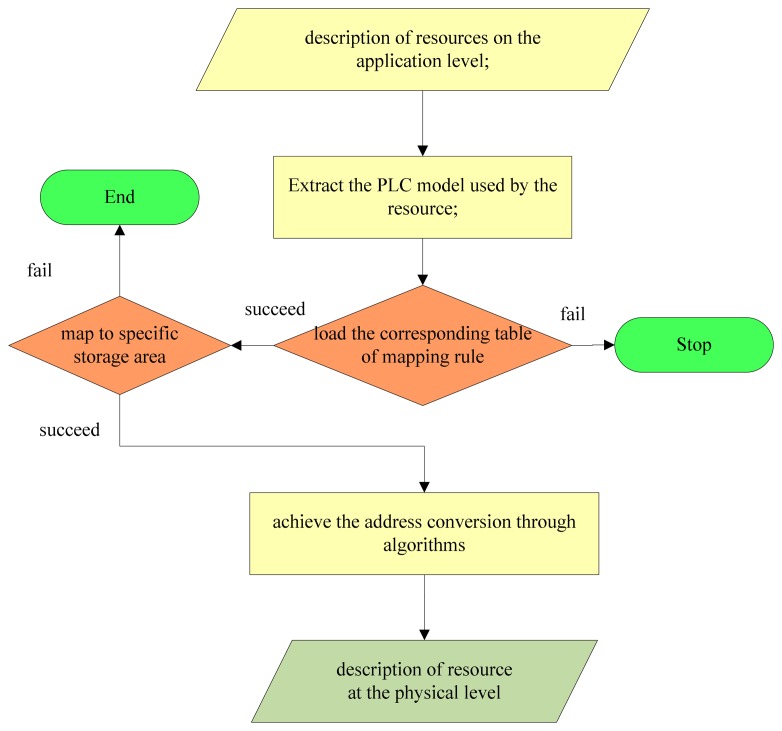
Diagram of the process for resources address mapping.

**Figure 4.**
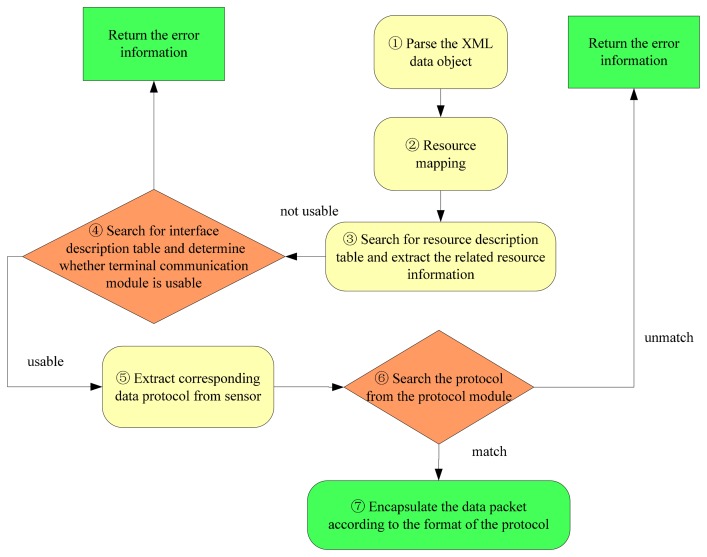
Procedures for data processing in resource management layer.

**Figure 5.**
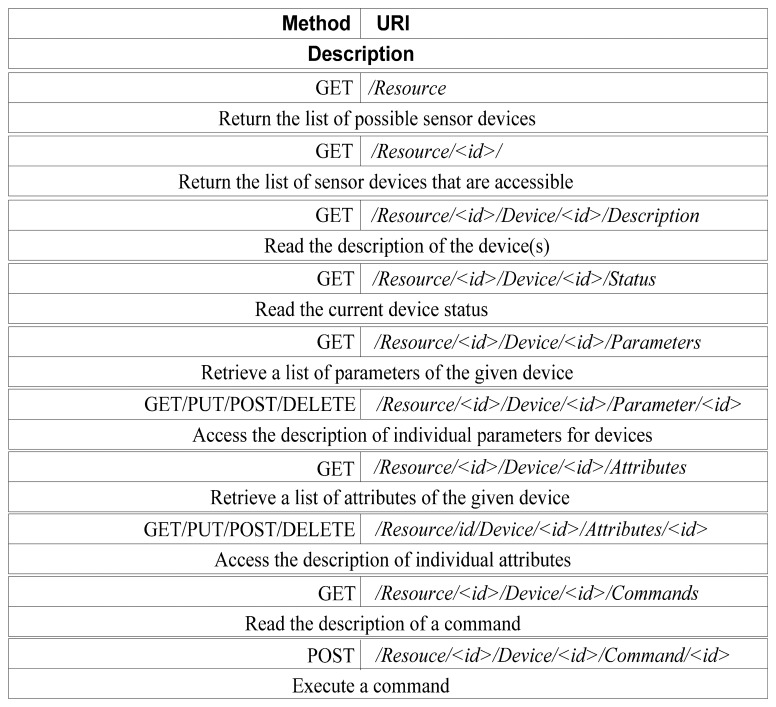
REST API for monitoring and controlling devices.

**Table 1.** Data packet conversion format for ASCII mode.

**STX**	**Start Character**
high address byte	Slave address
low address byte	

high level function code	Function code
low level function code	

data 1 high address	The first byte
data 1 low address	

……	……

Data *N* high address	The *N*-th Byte
Data *N* low address	

LRC high address	LRC
LRC low address	

End1	CR(0DH)

End2	LF(0AH)

**Table 2.** Data request packet for analog data.

**Send**			

**ASCII Mode**		**RTU Mode**	
	
**Field Name**	**Example (Hex)**	**Field Name**	**Example (Hex)**
Heading	3A	Slave Address	01
Slave Address	01	Function	03
Function	03	Starting Address Hi	06
Starting Address Hi	06	Starting Address Lo	14
Starting Address Lo	14	Number of Points Hi	00
Number of Points Hi	00	Number of Points Lo	08
Number of Points Lo	08	CRC(low byte)	04
Error Check(LRC)	DA	CRC(high byte)	80
End1(CR)	0D		
End2(LF)	0A		

**Table 3.** Data reply packet for analog data.

**Receive**			

**ASCII Mode**		**RTU Mode**	
	
**Field Name**	**Example (Hex)**	**Field Name**	**Example (Hex)**
Heading	3A	Slave Address	01
Slave Address	01	Function	03
Function	03	Bytes Count	10
Bytes Count	10	Data Hi (T20)	00
Data Hi (T20)	00	Data Lo (T20)	01
Data Lo (T20)	01	Data Hi (T21)	00
……	…	……	…
Data Lo (T27)	08	CRC (low byte)	72
Error Check (LRC)	C8	CRC (high byte)	98
End1 (CR)	0D		
End2 (LF)	0A		

**Table 4.** Basic protocol packets of the H7000.

**Data Packet ID**	**Type of Data Packet**
0 × 01	terminal request for registration
0 × 02	terminal request for cancellation
0 × 03	query of IP address for certain DTU
0 × 04	packet of invalid command or protocol (usually used for question answering or setup instructions)
0 × 05	receiving a data packet from a user (from DSC)
0 × 09	data packet from a user (from DTU to DSC)
0 × 0B	DTU parameters inquiry answer packet
0 × 0D	set up a DTU parameters inquiry answer packet

**Table 5.** Definition of resources.

**Number**	**Resource Name**
1	collection of sub-stations
2	specific sub-station
3	collection of PLCs in applications
4	specific PLC
5	collection of sensors
6	specific sensor

**Table 6.** Definition of resource labels.

**Resource Name**	**URL Label for Resources**
collection of sub-stations;	/stations
specific sub-station	/stations/{stationed}
all PLCs under sub-station	/stations/{stationed}/plcs
PLC under sub-station	/stations/{stationed}/plcs/{plcid}
sensor under certain PLC	/stations/{stationed}/plcs/{plcid}/{sensorid}

**Table 7.** Simple description of the resource interface.

**URL Description**	**Description**	**Description of Specific Function**
/stations	GET	list of information about all substations

/stations/{stationed}	GET	display the information for stationed
	PUT	update information about the stationid

/stations/{stationed}/plcs	GET	list information about all PLCs for stationid
	PUT	update information about all PLCs for stationid

/stations/{stationed}/plcs/{plcid}/{sensorid}	GET	display information about sensorid in plcid for stationid
	PUT	update information about sensorid in plcid for stationid

**Table 8.** Resource information comparison.

**Resource Name**	**Resource Method and ID**	**Examples**
read data from certain sensor	GET /stations/{stationed}/plcs/{plcid}/{sensorid}	http://localhost:8080/HeatingSystem/service/stations/1/1/D2000
write data to certain sensor	PUT /stations/{stationed}/plcs/{plcid}/{sensorid}/{value}	http://localhost:8080/HeatingSystem/service/stations/1/1/D2002/80.5

**Table 9.** Simple description of a resource operation table.

**Station ID**	**Plcid**	**Sensorid**	**Type**	**High**	**Low**
1	1	D2000	1		
1	1	D2002	2	58.5	98.5
1	2	D1000	1		
2	1	D1500	2	24.7	36.4

**Table 10.** Example I for the division of PLC storage area.

**Device**	**Range**		**Type**	**DVP (Hex)**	**Modbus (Decimal)**	**Effective**		

						**ES/EX/SS**	**SA/SX/SC**	**EH**
S	000∼255		bit	0000∼00FF	000001∼000256	0∼127	0∼1024	0∼1024
S	256∼511		bit	0100∼01FF	000257∼000512			
S	512∼767		bit	0200∼02FF	000513∼000768			
S	768∼1023		bit	0300∼03FF	000769∼001024			

X	000∼377(Octal)		bit	0400∼04FF	101025∼101280	0∼177	0∼177	000∼377
Y	000∼377(Octal)		bit	0500∼05FF	001281∼001536			

T	000∼255		bit	0600∼06FF	001537∼001792	0∼127	000∼255	000∼255
			word	0600∼06FF	401537∼401792			

M	000∼255		bit	0800∼08FF	002049∼002304	0∼1279	0∼4095	0000∼4095
M	256∼511		bit	0900∼09FF	002305∼002560			
M	512∼767		bit	0A00∼0AFF	002561∼002816			
M	768∼1023		bit	0B00∼0BFF	002817∼003072			
M	1024∼1279		bit	0C00∼0CFF	003073∼003328			
M	1280∼1535		bit	0D00∼0DFF	003329∼003584			
M	1536∼1791		bit	B000∼B0FF	045057∼045312			
M	1792∼2047		bit	B100∼B1FF	045313∼045568			
M	2048∼2303		bit	B200∼B2FF	045569∼045824			
M	2304∼2559		bit	B300∼B3FF	045825∼046080			
M	2560∼2815		bit	B400∼B4FF	046081∼046336			
M	2816∼3071		bit	B500∼B5FF	046337∼046592			
M	3072∼3327		bit	B600∼B6FF	046593∼046848			
M	3328∼3583		bit	B700∼B7FF	046849∼047104			
M	3584∼3839		bit	B800∼B8FF	047105∼047360			
M	3840∼4095		bit	B900∼B9FF	047361∼047616			

C	0∼199	16-bit	bit	0E00∼0EC7	003585∼003784	0∼127	0∼199	0∼199
			word	0E00∼0EC7	403585∼403784	0∼127	0∼199	0∼199

	200∼255	32-bit	bit	0EC8∼0EFF	003785∼003840	232∼255	200∼255	200∼255
			Dword	0EC8∼0EFF	403785∼403840	232∼255	200∼255	200∼255

**Table 11.** Structure of the mapping rule table for a Delta PLC.

**Storage Area**	**Starting Number (Decimal)**	**Ending Number (Decimal)**	**Hex Number**	**Heading (Hex)**
S	0	1023	10	0000
X	0	377	8	0400
Y	0	377	8	0500
T	0	255	10	0600
N	0	4095	10	0800
D	0	4095	10	1000
D	4096	9999	10	9000

**Table 12.** Structure of the resource status description table.

**Field Name**	**Definition**	**Comments**
NUMBER	number	number for resource access, starting from 1
COM_ID	Terminal ID	one resource corresponding to one number, and one number contains multiple resources
PLC_ID	ID for accessed PLC	one source belongs to one PLC hardware device
SENSOR_ID	Sensor ID	one sensor ID corresponding to one address in PLC
WORD_COUNT	word count for resource	1 or 2 means resource in analogue data, 0 means resource in digital switch
NAME	measure item	e.g., temperature, power
DEVICE_NAME	Device name	e.g., SIEMENS, DVP
DEVICE_PROTOCOL	Protocol name	MODBUS_RTU(Modbus protocol RTU mode) and MODBUS_ASC(Modbus protocol ASC mode)
DATA_TYPE	Data type	analog means analog data, digital means digital switch;
CONTROL_TYPE	Control type	number 1 means read only, 2 means writable
MEASURE_UNIT	Measure unit	e.g., Mpa: temperature (°C), pressure (Mpa)
VALUE	Measure value	means specific value
STATUS	status	0 means not usable, 1 means usable
FACTOR	factor	means original data need to determine factor
OFFSET	offset	means original data need to calculate offset

**Table 13.** Structure of the interface description table.

**Field Name**	**Definition**	**Comments**
COM_ID	communication ID	means ID for terminal communication module
PORT	port ID	means ID of the port access to the server
PROTOCOL	protocol	means GPRS protocol used by the terminal module
STATUS	status	0 means not usable, 1 means usable
OPEN	ON/OFF status;	0 means OFF, 1 means ON
